# Antiretroviral therapy as a risk factor for chronic kidney disease: Results from traditional regression modeling and causal approach in a large observational study

**DOI:** 10.1371/journal.pone.0187517

**Published:** 2017-12-07

**Authors:** Lise Cuzin, Pascal Pugliese, Clotilde Allavena, David Rey, Catherine Chirouze, Firouzé Bani-Sadr, André Cabié, Thomas Huleux, Isabelle Poizot-Martin, Laurent Cotte, Corinne Isnard Bagnis, Philippe Flandre, for the Dat’AIDS study group

**Affiliations:** 1 INSERM, UMR 1027, Toulouse, France; Université de Toulouse III, Toulouse, France; CHU Toulouse, COREVIH Toulouse, France; 2 Department of Infectious Diseases CHU Archet, Nice, France; 3 Department of Infectious Diseases CHU Hotel Dieu, Nantes, France; 4 HIV Infection Care Centre, Hôpitaux Universitaires, Strasbourg, France; 5 UMR CNRS 6249 Chrono-Environnement, Université de Franche-Comté; Service de maladies infectieuses, CHRU Besançon, France; 6 Reims Champagne-Ardenne University, Faculté de médecine, CHU Reims, Hôpital Robert Debré, Tropical and Infectious Diseases, Reims, France; 7 Departement of Infectious Diseases, INSERM CIC1424, Fort-de-France, France; 8 University Department of Infectious Diseases, CH Tourcoing, France; 9 Aix-Marseille University, Assistance Publique–Hôpitaux de Marseille—Hôpital Sainte-Marguerite, Immuno-hematology clinic, Marseille France, Inserm U912 (SESSTIM), Marseille, France; 10 Department of Infectious Diseases, Hospices Civils de Lyon, Lyon, France and INSERM U1052, Lyon, France; 11 Department of Nephrology, APHP and UPMC university, Paris, France; 12 Sorbonne Universités, UPMC Université, INSERM UMRS 1136, Institut Pierre Louis d’épidémiologie et de Santé Publique (IPLESP), Paris, France; Azienda Ospedaliera Universitaria di Perugia, ITALY

## Abstract

**Objective:**

We investigated whether patients receiving selected antiretroviral combinations had a higher risk of chronic kidney disease (CKD) using traditional regression modeling and a causal approach in a large prospective cohort.

**Patients and methods:**

For the purpose of this study, we selected 6301 patients who (i) started their first antiretroviral regimen after 1^st^ January 2004, (ii) had at least one serum creatinine measurement within 6 months before ART initiation (study entry), and (iii) had at least two measurements after study entry. Baseline eGFR was defined from the last serum creatinine measurement before study entry. All eGFR values were calculated using the Modification of Diet and Renal Disease (MDRD) equation. Both traditional Cox proportional hazards model and Cox marginal structural models were applied. Distinct coding for antiretroviral therapy exposure were investigated as well as double robust estimators.

**Results:**

Overall we showed that patients receiving tenofovir (TDF) with a ritonavir boosted protease inhibitor (rbPI) exhibited a higher risk of CKD compared with patients who received TDF with a non-nucleosidic reverse transcriptase inhibitor (NNRTI). Such an increased risk was observed considering both initial and current regimens. Our analysis revealed a clinician-driven switch away from TDF among persons experiencing a decline in renal function while receiving this drug.

**Conclusion:**

Our results show that combination of TDF and boosted protease inhibitor is associated with a higher CKD risk than TDF and a NNRTI.

## Introduction

Patients living with HIV have an increased risk of chronic kidney disease (CKD) by comparison with the general age-matched population, for various reasons including the use of some antiretroviral agents (ARVs) [1, 2]. ARVs have been estimated to be responsible for 0.5–14% of all cases of renal impairment in these patients [3, 4].

Many studies aimed to investigate CKD risk factors, including both traditional renal risk factors and ARVs. Summarizing the information is complex due to the various studied populations, endpoints, statistical methods and variables coding. The studied population may concern all HIV-infected individuals with at least two estimated glomerular filtration rates (eGFR) measurements or be restricted to individuals with baseline eGFR>60 ml/min/1.73m^2^ or >90 ml/min/1.73m^2^ [5–9]. Outcomes may be CKD stage 3a (<60 ml/min/1.73m^2^), CKD stage 3b (<45 ml/min/1.73m^2^), CKD stage 4 (<30 ml/min/1.73m^2^), or a change in the eGFR slope (pre-ART versus on ART) [5, 6, 10]. Different statistical methods were used to investigate the association between ART exposure and occurrence of CKD, with differences in the assessment of the association between CKD and ART exposure, including different coding of ART exposure (current use versus past use, cumulative exposure) [7, 9]. Furthermore, the lack of randomization in observational studies and the frequent modifications of ART during the patients’ follow-up are challenging when searching the relationship between exposure to some ARVs and the risk of CKD. Notably, it has been showed that treatment discontinuation of specific ARVs was related to the current eGFRs, indicating that such a marker should be treated as a time-varying confounder [6, 7]. Some authors have only considered the CKD risk in relation with the initial ARV regimen [10], whether or not patients remained on this regimen, whereas others investigated current/past ARV exposure or cumulative exposure [5–7, 11]. Analyses using marginal structural models (MSM) censored patients upon any change of ARV, ensuring an appropriate adjustment for confounding factors but limiting the number of events to those occurring while patients received their original ARV regimen [10, 12].

Some studies have shown that regimens including tenofovir (TDF) in association with a ritonavir boosted protease inhibitor (rbPI) were related with greater eGFR decline by comparison with regimens based on TDF and a non-nucleosidic reverse transcriptase inhibitor (NNRTI). Unfortunately, those studies involved relatively small numbers of patients and did not investigate the occurrence of CKD [13–15]. A recent study investigated exposure to TDF in association with a rbPI or with a NNRTI as risk factors for CKD events (stages 3 or 4) but provided conflicting results [10]. Initial regimens with TDF plus a rbPI were significantly associated with an increased risk of CKD using MSM but not using a Cox proportional hazards model [10]. The former analysis considered only CKD events occurring while individuals were still receiving their initial regimen, censoring patients upon any change in ARV, whereas the latter analysis used all events but considered only the initial regimen. A significant improvement in the eGFR slope while receiving TDF plus a rbPI has even been observed [10]. From all these studies, it is quite difficult to have a clear idea of the impact of a regimen containing TDF in association with a rbPI on the risk of CKD and furthermore to draw any conclusions regarding the causality.

Our aim was to evaluate ARV exposure as a risk factor for CKD (defined by an eGFR below 60 ml/min/1.73m^2^) using different statistical considerations applied to a large prospective cohort of patients living with HIV. We investigated the risk of CKD in patients receiving a combination with or without TDF in association either with a rbPI or a NNRTI. CKD risk was investigated in relation with the initial regimen for all events occurring during the follow-up or only for those occurring during the initial regimen.

## Patients and methods

### Patients

Information was collected from 12 centers in France participating in the Dat’AIDS cohort (Clinicaltrials.gov ref NCT02898987) after approval by French National Committee on Informatics and Human Rights (CNIL n°1357652) [16]. The authors did not have access to patient identifying information as part of this work. For the purpose of this study, we selected patients who started their first antiretroviral regimen (ART) after 1^st^ January 2004, had at least one serum creatinine measurement within 6 months before ART initiation which was >60ml/min/1.73m^2^, and at least two measures after ART initiation. Baseline eGFR was defined from the last serum creatinine measurement before ART initiation. All eGFR values were calculated using the Modification of Diet and Renal Disease (MDRD) equation. Other baseline values were defined at or before ART initiation.

### Outcomes and ARV exposure

CKD event was defined by a confirmed (3 months apart) eGFR below 60 ml/min/1.73m^2^. Time to event was the time between ART initiation and the first eGFR below 60 ml/min/1.73m^2^. Antiretroviral regimens were categorized into five mutually exclusive combinations: TDF combined with a rbPI (TDF+/rbPI); TDF combined with a NNRTI (TDF+/NNRTI); a rbPI without TDF (TDF-/rbPI); a NNRTI without TDF (TDF-/NNRTI); and any other combination with or without TDF (Other ART). The “other” regimen was only used to adjust for other types of regimens and no hazard ratios are provided. Initial, current or past regimens were classified according to these categories. Past regimens included only the regimens immediately prescribed before the current regimen.

### Objectives

Our main objective was to determine whether patients receiving a regimen of TDF+/rbPI had a greater risk of developing CKD by comparison with patients receiving other regimens, in particular those receiving TDF+/NNRTI. We also compared results obtained using different statistical methods corresponding to different analyses and objectives. We conducted two analyses as to examine the effect of ART exposure on the risk of CKD. The first analysis was based on a series of standard proportional hazard models involving all CKD events occurring during the follow-up. Different ways of exploring the association between regimen-types and CKD were investigated. All those models were adjusted for baseline and time-varying variables introduced in the next section. The second analysis examined only the association between the initial regimen type and CKD occurring while patients received this first regimen, censoring patients upon any change in ART. This latter analysis involved both standard Cox regression models and marginal structural proportional hazards model based on a causal approach. Double robust estimators were also provided to prevent potential model misspecification [17–19].

### Potential confounders

Potential baseline confounding factors were age, CD4 cell count nadir, CD4 cell count, viral load (HIV-1 RNA), eGFR, year of ART initiation, gender, HIV exposure (homosexual vs. heterosexual vs. other), HCV or HBV co-infections, prior diabetes, hypertension and cardiovascular disease (CVD). Prior diabetes, hypertension and CVD were considered present if notified in the patient’s medical history or if the patient’s prescriptions contained any drugs in relation with these diseases.

Standard proportional hazards models were adjusted for baseline confounders and also for time-varying CD4 cell count and viral load using the last value-carried-forward method. In the causal approach, eGFR measured at different visits was also used as time-varying confounders of the relationship between ART exposure and both censoring and switch from the initial regimen.

### Models

A first series of standard proportional hazards models was fitted to the dataset. These models were used to examine the relationship between regimen-type exposure with CKD adjusted for baseline and time-varying variables. In this first series the number of events is the total number of CKD occurring during follow-up. A first model investigated the association between occurrence of CKD with initial regimen-type, a second model considered the regimen-type as a time-varying covariate (current regimen-type), and a third model used both current and past regimen-types. All patients were included in these analyses whether or not they remained on their initial ART regimen.

Propensity scores and Marginal Structural Models (MSM) have been introduced to overcome the lack of randomization in observational studies [12, 20, 21]. In MSM analyses patients are censored upon any change in ART limiting the number of events to those occurring while patients received their first ART regimen. Fitting these models involved estimation of Inverse Probability of Treatment Weighting (IPTW) using propensity score.

Doubly robust estimation combines outcome regression with weighting by the propensity score such that the effect estimator is robust to misspecification of one of these two models [17, 19, 22]. In our study, we adjusted the weighted Cox model with baseline and time-varying covariates to provide estimates having doubly robust property [18, 23]. Estimation of stabilized IPTW is described in the S1 File.

Negative binomial models were used to investigate ART regimen-type discontinuation rates in relationship to the current eGFR level [6]. No adjustment for multiple testing was carried out.

### Data analysis

Baseline and time-varying continuous variables were explored in different ways including linear terms, categories, cubic and quadratic spline with four knots. In particular, we followed the recommendations and guidelines of Cole and Hernan for constructing IPTW [24]. IPTW were estimated with polytomous logistic models and linear terms for continuous variables provided the best stabilized weights SW^A^.

Each person’s follow-up was divided into a series of consecutive 1-month periods and each person’s covariate data (CD4 cell count, HIV-1 RNA and eGFR) was also updated at the start of each month [20]. Pooled logistic regression models were fitted to such data providing estimates of corresponding weights for censored and switching away observations (see appendix for details). Restricted cubic splines with three knots (10^th^, 50^th^ and 90^th^ percentiles) for the month since study entry were used for a time-dependent intercept [20]. Continuous baseline variables (age, CD4 cell count nadir, CD4 cell count, HIV-1 RNA, eGFR) were also included with cubic three-knot splines in pooled logistic models and year of regimen-type as a linear term. Time-varying CD4 (>350, 199–350 and < = 200 cells/mm^3^), viral load (HIV-1 RNA < = 50, 51–500 and >500 copies/mL) and eGFR (< = 50, 51–70, 71–90, >90 ml/min/1.73m^2^) were included as step function (categories) in pooled logistic models. The mean of the final stabilized weights SW(t) were 0.998 (standard deviation, 0.331) with a range of 0.33 to 3.73. These weights are shown over follow-up in S1 Fig.

## Results

Baseline characteristics of the 6 301 included patients are described in Table 1. Most patients started their ART with a rbPI-containing regimen combined with TDF (N = 2 360, 37%) or without TDF (N = 1 606, 25%). Patients had a median of 12 (interquartile range (IQR), 6–20) creatinine measurements during follow-up with a median of 2.6 months interval (IQR: 1.1–3.8 months) between two consecutive measurements. Patients who started ART with TDF-/NNRTI had a lower baseline eGFR than patients receiving another regimen (p<0.001) whereas patients starting with TDF+/NNRTI had both a higher nadir CD4 and higher baseline CD4 than patients receiving another regimen (p<0.001). Overall, the cumulative follow-up duration on any ART regimen-types was 22 297 person-years with a median of 3.1 years (IQR: 1.5–5.2 years).

**Table 1:** Baseline characteristics of the studied population.

		All	TDF+/NNRTI	TDF+/rbPI	TDF-/NNRTI	TDF-/rbPI	Other
N (%)		6301	1183 (19)	2360 (37)	285 (5)	1606 (25)	867 (14)
Age (years) at ART initiation, median (IQR)		39 (32–47)	38 (32–46)	40 (32–47)	39 (32–48)	37 (30–46)	40 (33–48)
Baseline eGFR (ml/min/1.73m^2^), median (IQR)		101 (86–118)	102 (87–117)	102 (88–119)	91 (81–110)	100 (84–121)	100 (87–117)
Baseline CD4 count (cells/mm3), median (IQR)		289 (175–392)	325 (245–430)	276 (147–385)	272 (207–331)	275 (164–373)	298 (144–413)
Baseline HIV-1 viral load (log_10_.copies/ml), median (IQR)		4.8 (4.2–5.3)	4.7 (4.1–5.1)	4.9 (4.4–5.4)	4.8 (4.3–5.2)	4.8 (4.2–5.3)	4.8 (4.2–5.3)
Nadir CD4 count (cells/mm^3^), median (IQR)		264 (148–358)	304 (218–391)	253 (130–359)	251 (164–314)	246 (132–338)	268 (119–366)
Gender	Male, n (%)	4583 (73)	939 (79)	1813 (77)	212 (74)	967 (60)	652 (75)
HIV exposure	Homosexual, n (%)	2812 (45)	646 (55)	1122 (48)	119 (42)	527 (33)	398 (46)
	Heterosexual, n (%)	2803 (45)	422 (36)	1000 (42)	139 (49)	905 (56)	337 (39)
	Other or unknown, n (%)	686 (11)	115 (10)	238 (10)	27 (10)	174 (11)	132 (15)
Hepatitis B, n (%)		317 (5)	60 (5)	159 (7)	6 (2)	39 (2)	53 (6)
Hepatitis C, n (%)		459 (7)	72 (6)	192 (8)	19 (7)	97 (6)	79 (9)
Hypertension, n (%)		247 (4)	44 (4)	77 (3)	14 (5)	79 (5)	33 (4)
CVD, n (%)		102 (2)	14 (1)	41 (2)	9 (3)	20 (1)	18 (2)
Diabetes, n (%)		113 (2)	21 (2)	30 (1)	10 (4)	33 (2)	19 (2)
rbPI or NNRTI component of initial ART regimen, n (%)							
	Efavirenz		989 (84)		172 (30)		
	Nevirapine		95 (8)		106 (37)		
	Etravirine		14 (1)		7 (2)		
	Rilpivirine		85 (7)		0		
	rDarunavir			911 (39)		258 (16)	
	rAtazanavir			669 (28)		251 (16)	
	rLopinavir			626 (27)		855 (53)	
	rFosamprenavir			101 (4)		169 (11)	
	rSaquinavir			50 (2)		43 (3)	
	rTipranavir			3 (0)		0	

**Fig 1:**
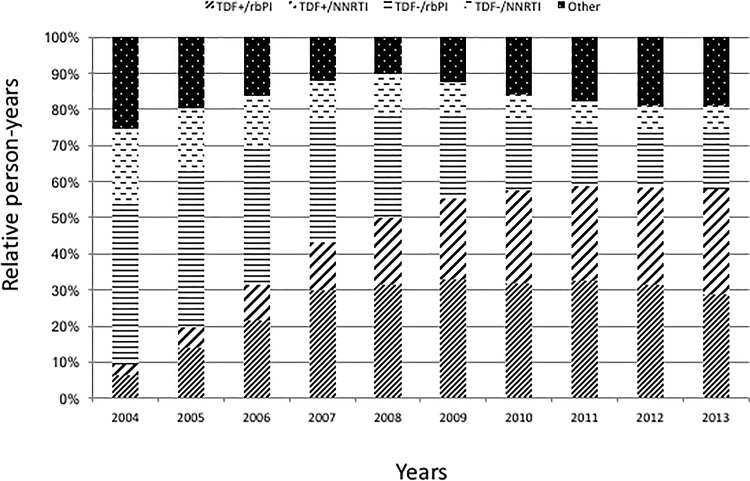
Relative proportions of person-years receiving the studied regimens.

Overall, 2 963 (47%) patients remained on their initial regimen throughout the follow-up, including 48 and 66% of the patients who received initially TDF+/rbPI or TDF+/NNRTI, respectively, and 37% and 34% of the patients who received initially a TDF-/rbPI or TDF-/NNRTI regimen respectively. The median number of regimens received was 2 (IQR: 1–2) and only 610 (10%) patients received 3 or more regimens in addition to the initial regimen. From 2004 to 2013, the relative proportion of person-years receiving a regimen without TDF decreased from 65 to 23%. In contrast, the relative proportion receiving a TDF-containing regimen increased from 9 to 58%, especially with a NNRTI, from 3 to 29% (Fig 1).

**Fig 2:**
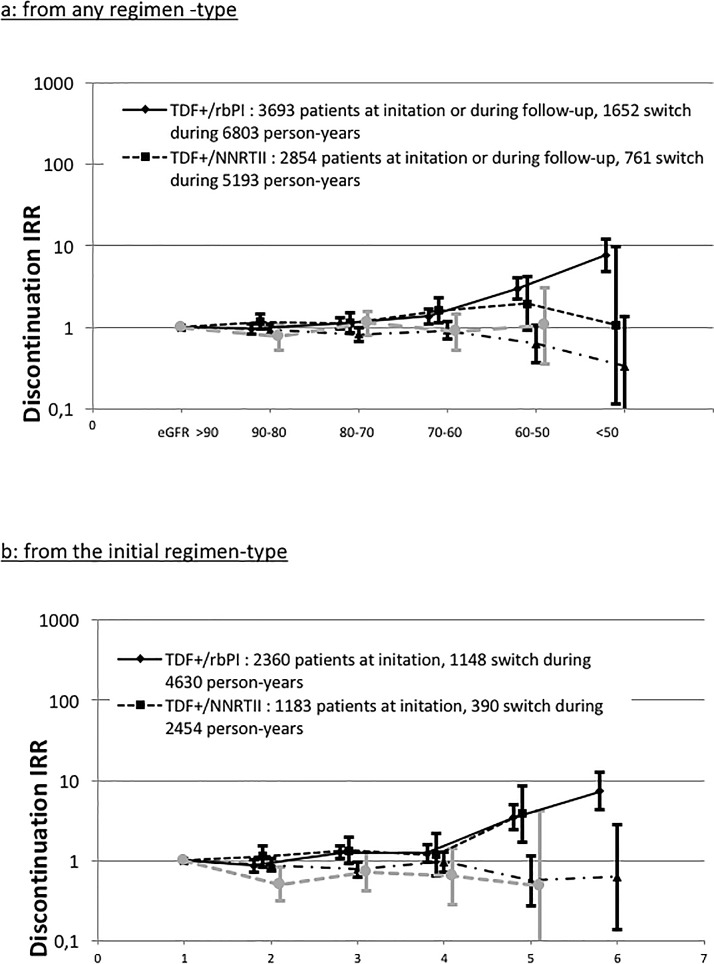
Adjusted incidence rate ratio of switching away from regimen-types during the follow-up according to current eGFR. Panel a. from any regimen–type. Panel b: from the initial regimen-type.

Incidence rate ratio, adjusted for baseline covariates, of switching away from current regimen-types during follow-up significantly increased as eGFR declined for the TDF+/rbPI regimen (Fig 2A). Considering only switches from the initial regimen, this increase was observed for patients receiving TDF-containing regimens with a rbPI or with a NNRTI (Fig 2B).

Occurrence of CKD was observed in 211 (3.4%) patients, corresponding to an incidence of 9.6 (95% confidence interval (CI), 8.3–10.9) cases per 1000 person-years. Among those, 87 (41%) patients developed CKD while on their initial ART regimen, incidence of 6.9 (95% CI, 5.4–8.3) cases per 1 000 person-years. Of note, only 31 (0.5%) and 9 (0.1%) patients developed CKD stage 3b defined with a confirmed eGFR below 45 and 30 ml/min/1.73m^2^, respectively.

All comparisons in both Tables 2 and 3 are made with patients receiving TDF+/NNRTI as the reference group. Table 2 presents Hazard Ratios (HR) from analyses considering all CKD events occurring in the studied population. Initial regimen with TDF+/rbPI was significantly associated with an increased CKD risk in model 1 (HR = 2.15, 95% CI, 1.3 to 3.6). When the regimen-type was used as a time-dependent variable in model 2 all ART regimens were associated with an increased CKD risk compared with patients receiving TDF+/NNRTI. A further adjustment with the previous regimen-type received before the current regimen provides similar HR for the current regimens. However, TDF-/NNRTI as past regimen was associated with a lower risk of CKD although not statistically significant.

**Table 2:** Hazard ratios for all CKD events.

		Hazard ratio (95%CI)	p
		211 events	
**Model 1: Initial ARV regimen**			
TDF+/rbPI vs. TDF+/NNRTI		2.15 (1.3–3.6)	0.004
TDF-/NNRTI vs. TDF+/NNRTI		1.14 (0.5–2.4)	0.74
TDF-/rbPI vs. TD-+/NNRTI		1.27 (0.7–2.2)	0.41
**Model 2: Current ARV regimen**			
TDF+/rbPI vs. TDF+/NNRTI		1.73 (1.1–2.8)	0.03
TDF-/NNRTI vs. TDF+/NNRTI		2.10 (1.1–3.9)	0.02
TDF-/rbPI vs. TDF+/NNRTI		1.90 (1.2–3.1)	0.01
**Model 3**			
**Current ARV regimen**			
TDF+/rbPI vs. TDF+/NNRTI		1.79 (1.1–2.9)	0.02
TDF-/NNRTI vs. TDF+/NNRTI		2.50 (1.3–4.8)	0.01
TDF-/rbPI vs. TDF+/NNRTI		1.97 (1.2–3.3)	0.01
**Previous ARV regimen**			
TDF+/rbPI		2.79 (1.8–4.2)	< .0001
TDF+/NNRTI		1.68 (0.9–3.0)	0.08
TDF-/NNRTI		0.62 (0.2–1.8)	0.36
TDF-/rbPI		1.30 (0.8–2.1)	0.31

All models were adjusted for age at initiation, CD4 cell count nadir, baseline CD4 cell count, baseline viral load (HIV-1 RNA), baseline eGFR, year of ART initiation, gender, HIV exposure (homosexual vs. heterosexual vs. other), HCV or HBV co-infections, prior diabetes, prior hypertension, prior cardiovascular disease, time-varying CD4 cell count and time-varying viral load

Results of analyses of the risk of CKD occurring while patients were still receiving their initial regimen are summarized in Table 3. The unadjusted standard Cox model indicated a higher risk of CKD in patients receiving TDF+/rbPI compared with those receiving TDF+/NNRTI (HR = 3.14, 95%CI, 1.4 to 7.0). TDF-/rbPI was also associated with a higher CKD risk (HR = 2.38, 95% CI, 1.0 to 5.5).

**Table 3:** Hazard ratio for CKD occurring during the initial regimen type.

	Hazard ratio (95%CI)	p
**Crude model (unadjusted)**	87 events	
TDF+/rbPI vs. TDF+/NNRTI	3.14 (1.4–7.0)	0.005
TDF-/NNRTI vs. TDF+/NNRTI	1.22 (0.3–4.8)	0.77
TDF-/rbPI vs. TDF+/NNRTI	2.38 (1.0–5.5)	0.04
**Adjusted model** *		
TDF+/rbPI vs. TDF+/NNRTI	3.07 (1.3–7.1)	0.009
TDF-/NNRTI vs. TDF+/NNRTI	1.26 (0.3–5.2)	0.74
TDF-/rbPI vs. TDF+/NNRTI	2.06 (0.8–5.0)	0.11
**Causal approach**		
**Weighted model**		
TDF+/rbPI vs. TDF+/NNRTI	2.71 (1.3–5.6)	0.007
TDF-/NNRTI vs. TDF+/NNRTI	1.18 (0.3–4.4)	0.81
TDF-/rbPI vs. TDF+/NNRTI	2.13 (1.0–4.6)	0.05
**Double robust estimators**		
TDF+/rbPI vs. TDF+/NNRTI	3.25 (1.5–6.9)	0.002
TDF-/NNRTI vs. TDF+/NNRTI	1.18 (0.3–4.5)	0.81
TDF-/rbPI vs. TDF+/NNRTI	2.08 (0.9–4.6)	0.07

*All model were adjusted for age at initiation, CD4 cell count nadir, baseline CD4 cell count, baseline viral load (HIV-1 RNA), baseline eGFR, year of ART initiation, gender, HIV exposure (homosexual vs. heterosexual vs. other), HCV or HBV co-infections, prior diabetes, prior hypertension, prior cardiovascular disease, time-varying CD4 cell count and time-varying viral load

Adjusting models with baseline and time-varying confounders show similar HR estimates although not statistically significant for patients receiving TDF-/rbPI. Weighted and double robust models, based on our causal approach, show that patients who received TDF+/rbPI had still a higher risk of CKD than those treated with TDF+/NNRTI (HR = 2.71, 95% CI, 1.3 to 5.6 and HR = 3.25, 95% CI, 1.5 to 6.9). Patients who received TDF-/rbPI have also a higher risk of CKD though marginally significant.

## Discussion

We showed that patients receiving tenofovir in association with a ritonavir boosted protease inhibitor exhibited a higher risk of CKD by comparison with patients receiving tenofovir in association with a non-nucleosidic reverse transcriptase inhibitor. This result was found in all our analyses, whatever the statistical methods, both for the CKD events occurring while patients received their initial regimen and for the CKD events observed during the follow-up. Rates of discontinuation in patients receiving a TDF-containing regimen increased as current eGFR decreased, indicating clearly that eGFR is a time-varying confounder in the relationship between CKD and ART exposure.

Several studies suggested a higher risk of CKD in patients receiving TDF in combination with a rbPI by comparison with patients receiving TDF with a NNRTI [10, 13, 14]. Those studies, however, included a limited number of patients and, for some of them, did not involve the most recent rbPIs. One study used marginal structural models but did not involve current eGFR as a time-varying confounder in computing stabilized weights. They displayed conflicting results [10]. Previous studies provided incidence rates of CKD varying from 6.2 to 10.6 cases per 1000 person-years, our estimate of 9.6 cases per 1000 person-years is in agreement with those results [8–10]. In our study, incidences of CKD were 6.9 cases per 1000 person-years when considering only CKD occurring while patients still received their initial ART regimen.

Our study has some limits. We used MDRD estimations of GFR because our available data did not allow the use of Cockroft-Gault formula nor Chronic Kidney Disease-Epidemiology Collaboration (CKD-EPI) equation, which is currently recommended by the latest guidelines of the Infectious Diseases Society of America [25]. Not all NNRTIs nor bPIs have the same impact on renal function, and TDF+/NNRTI are mostly efavirenz containing regimens (84%) whereas only 30% of the TDF-NNRTI contained efavirenz. This could be a major source of confounding. But we kept on with those regimens classes because of the small number of events.

The first series of analyses based on standard proportional hazard models provided different results according to how ART exposure was coded in models, corresponding to different objectives. Approximately 60% of CKD occurred after patients moved from their initial regimen. Thus, considering only the initial regiment-type captures only a part of the relationship between ART exposure and risk of CKD. This has been done in the study published by Kalayjian and colleagues [10]. It should be considered with caution although it has been shown that a decline in the eGFR during TDF therapy was not always reversible [26]. Our results, like other studies, show an increasing discontinuation rate for some antiretroviral regimens including tenofovir, alongside observed eGFR decrease [6, 7].

The use of current ART exposure as a time-varying variable in standard proportional hazard models indicated that all regimen-types are associated with an increased CKD risk compared with TDF+/NNRTI. Omission of variables summarizing the history of ART exposure in that model likely explained such results. Some CKD events occurred after many years of ARV exposure. This can be explained by the long-term effect of ARV on CKD risk [27]. Addition of the previous regimen in the model showed that TDF-/NNRTI was associated with a lower CKD risk, though not statistically significant. The association between TDF-/NNRTI as current regimen and the increased risk of CKD is surprising and questioned the use of such coding in Cox models.

Analyses based on our causal approach, though limited to a relatively small number (87) of CKD events, show an association between an initial regimen based on TDF+/rbPI and an increased risk, by comparison with initial regimens based on TDF+/NNRTI. Considering only CKD events occurring while patients were still receiving their initial regimen, all models provide similar findings.

## Conclusions

In conclusion, we observed a higher risk of CKD in patients receiving TDF+rbPI, compared with patients receiving TDF+NNRTI. Incidence rates of CKD found in our work are in agreement with those found in other studies. Current eGFR is clearly shown as a prognostic factor of switching away from a TDF-containing regimen. Consequently, e-GFR should be then taking into account in estimated stabilized weights for marginal structural models and double robust estimators.

This research did not receive any specific grant from funding agencies in the public, commercial, or not-for-profit sectors.

## Supporting information

S1 File IPTW calculation. (DOCX)

S1 Fig Box plots of stabilized weights. (DOCX)
