# Supplementary material for: Antiretroviral therapy as a risk factor for chronic kidney disease: Results from traditional regression modeling and causal approach in a large observational study
Source: PLoS One. 2017 Dec 7;12(12):e0187517. doi: 10.1371/journal.pone.0187517 (PMC5720720; doi:10.1371/journal.pone.0187517)
Supplement: S1 File — (DOCX) [file pone.0187517.s001.docx]

Antiretroviral therapy as a risk factor for chronic kidney disease: causal approach.

Lise Cuzin (MD)^a^, Pascal Pugliese (MD)^b^, Clotilde Allavena (MD)^c^, David Rey (MD)^d^, Catherine Chirouze (MD, PhD)^e^ , Firouzé Bani-Sadr (MD, PhD)^f^, André Cabié (MD, PhD)^g^, Thomas Huleux (MD)^h^, Isabelle Poizot-Martin (MD)^i^, Laurent Cotte (MD)^j^, Corinne Isnard Bagnis (MD, PhD)^k^, Philippe Flandre (PhD)^l^ for the Dat’AIDS study group†

Supplemental material : IPW calculation

Inverse probability weights

N is the number of patients in the corresponding population, *i*=1, 2, …, N. Let A denote the regimen-type received at baseline with A=1 to 5 (TDF+/rbPI, TDF+/NNRTI, TDF-/rbPI, TDF-/NNRTI and Other ART) with reference regimen-type TDF-/rbPI. Let T be the time in years from study entry to occurrence of CKD, C is the time in years from study entry to censoring due to either administrative censoring or lost to follow-up and S is the time in years from study entry to switch from the initial regimen without experiencing the CKD event. Let D_1i_(t) be an indicator that participant *i* is censored artificially or due to loss of follow-up at time *t*, D_2i_(t) be an indicator that participant *i* switched from his initial regimen to another regimen-type before experiencing the CKD event at time *t*. Finally, let L(t) be a vector of time-fixed and time-varying confounders while V is a vector subset of L(t) corresponding to time-fixed confounders measured at study entry.

The following stabilized weights were used to fit marginal structural proportional hazards model: $SW\left( t \right)={SW}^{A} {SW}^{C_{1}}\left( t \right) {SW}^{C_{2}}\left( t \right)$, where SW^A^ is the inverse probability of treatment weights (IPTW), ${SW}^{D_{1}}\left( t \right)$is the inverse probability of censored weights (IPCW) and ${SW}^{D_{2}}\left( t \right)$ is the inverse probability of switching weights (IPSW), considering only switches from the initial regimen-type.

IPTW for the five regimen-types are defined as follows: ${SW}^{A}=\Pr\left[ A=j \right]/Pr[A=j|V]$, where $\Pr\left[ A=j \right]$is the marginal proportion of patients receiving the regimen-type *j* (*j*=1,2, …, 5) in the study sample, $Pr[A=j|V]$ is the proportion of patients receiving the regimen-type *j* conditional on the vector V measured at study entry. ${SW}^{C_{1}}\left( t \right)$ and ${SW}^{C_{2}}\left( t \right)$ are defined as follows:

$${SW}^{C_{1}}(t)=\prod_{u\leq t} \frac{\Pr\left[ D_{1}\left( u \right)=0 \right|V, D_{1}\left( u^{-} \right)=0,D_{2}\left( u^{-} \right)=0,T\geq u]}{\Pr\left[ D_{1}\left( u \right)=0 \right|L\left( u \right), D_{1}\left( u^{-} \right)=0,D_{2}\left( u^{-} \right)=0,T\geq u]}$$

$${SW}^{C_{2}}(t)=\prod_{u\leq t} \frac{\Pr\left[ D_{2}\left( u \right)=0 \right|V, D_{2}\left( u^{-} \right)=0,D_{1}\left( u^{-} \right)=0,T\geq u]}{\Pr\left[ D_{2}\left( u \right)=0 \right|L\left( u \right), D_{2}\left( u^{-} \right)=0,D_{1}\left( u^{-} \right)=0,T\geq u]}$$

where *u*^-^ indicates the time before time *u.*

SW^A^ are estimated once at initiation of the initial regimen-type with a polytomous logistic regression whereas both SW^C^(t) and SW^S^(t) are estimated using pooled logistic regression along the follow-up [1-3].

1. D'Agostino RB, Lee ML, Belanger AJ, Cupples LA, Anderson K, Kannel WB. Relation of pooled logistic regression to time dependent Cox regression analysis: the Framingham Heart Study. Statistics in medicine. 1990;9(12):1501-15. PubMed PMID: 2281238.

2. Hernan MA, Brumback B, Robins JM. Marginal structural models to estimate the causal effect of zidovudine on the survival of HIV-positive men. Epidemiology. 2000;11(5):561-70. PubMed PMID: 10955409.

3. Howe CJ, Cole SR, Mehta SH, Kirk GD. Estimating the effects of multiple time-varying exposures using joint marginal structural models: alcohol consumption, injection drug use, and HIV acquisition. Epidemiology. 2012;23(4):574-82. doi: 10.1097/EDE.0b013e31824d1ccb. PubMed PMID: 22495473; PubMed Central PMCID: PMC3367098.
