# Supplementary material for: Antiretroviral therapy as a risk factor for chronic kidney disease: Results from traditional regression modeling and causal approach in a large observational study
Source: PLoS One. 2017 Dec 7;12(12):e0187517. doi: 10.1371/journal.pone.0187517 (PMC5720720; doi:10.1371/journal.pone.0187517)

Antiretroviral therapy as a risk factor for chronic kidney disease: causal approach.

Lise Cuzin (MD)^a^, Pascal Pugliese (MD)^b^, Clotilde Allavena (MD)^c^, David Rey (MD)^d^, Catherine Chirouze (MD, PhD)^e^ , Firouzé Bani-Sadr (MD, PhD)^f^, André Cabié (MD, PhD)^g^, Thomas Huleux (MD)^h^, Isabelle Poizot-Martin (MD)^i^, Laurent Cotte (MD)^j^, Corinne Isnard Bagnis (MD, PhD)^k^, Philippe Flandre (PhD)^l^ for the Dat’AIDS study group†

SM Figure 1: Box plots of stabilized weights


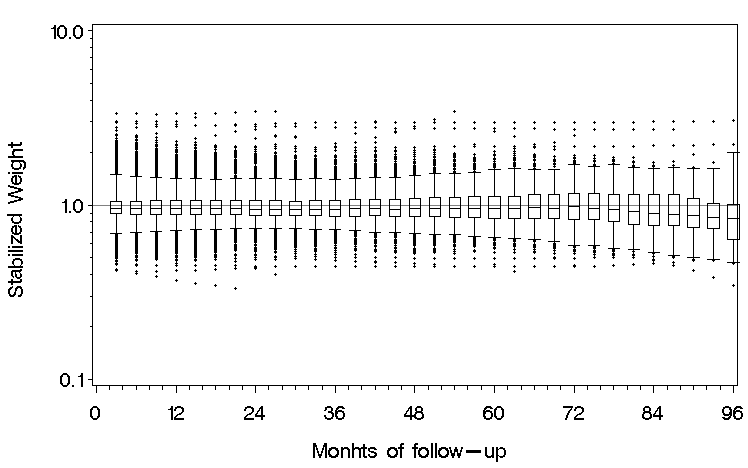

Supplement: S1 Fig — (DOCX) [file pone.0187517.s002.docx]
